# Impact of COVID-19 pandemic on the daily management of biotechnological therapy in inflammatory bowel disease patients: Reorganisational response in a high-volume Italian inflammatory bowel disease centre

**DOI:** 10.1177/2050640620929133

**Published:** 2020-05-21

**Authors:** Franco Scaldaferri, Daniela Pugliese, Giuseppe Privitera, Sara Onali, Loris Riccardo Lopetuso, Gianenrico Rizzatti, Carlo Romano Settanni, Marco Pizzoferrato, Elisa Schiavoni, Laura Turchini, Valeria Amatucci, Daniele Napolitano, Tiziana Bernabei, Vincenzina Mora, Lucrezia Laterza, Alfredo Papa, Luisa Guidi, Gian Lodovico Rapaccini, Antonio Gasbarrini, Alessandro Armuzzi

**Affiliations:** 1 CEMAD – IBD UNIT – Unitá Operativa Complessa di Medicina Interna e Gastroenterologia, c Fondazione Policlinico Universitario “A. Gemelli” IRCCS, Rome, Italy; 2 Dipartimento Universitario di Medicina e Chirurgia Traslazionale, Università Cattolica del Sacro Cuore, Rome, Italy

**Keywords:** COVID-19, inflammatory bowel disease centre, organisational impact, clinical impact, biological therapy

## Abstract

The coronavirus disease 2019 (COVID-19) pandemic is having a major clinical as well as organisational impact on the national health-care system in Italy, particularly in high-volume hospitals which are usually active for many essential clinical needs, including inflammatory bowel disease (IBD). Here, we report major clinical and organisational challenges at a high-volume Italian IBD centre one month after the start of the Italian government’s restrictions due to the COVID-19 pandemic. All routine follow-up IBD visits of patients in remission were cancelled or rescheduled for 8–12 weeks’ time. However, access to the hospital for therapy or for unstable/relapsing patients was not considered postponable. Everyone attending the centre (e.g. physicians, nurses, administrative personnel and patients) were advised to respect the general recommended rules for hand hygiene and social distancing, to disclose if they had a fever or cough or flu-like symptoms and to wear a surgical mask and gloves. At the entrance of the therapy area, a control station was set up in order to double-check all patients with a clinical interview and conduct thermal scanning. A total of 1451 IBD patients under biotechnological or experimental therapy actively followed in the CEMAD IBD centre were included in the study. About 65% of patients maintained their appointment schedules without major problems, while in 20% of cases planned infusions were delayed because of the patient’s decision or practical issues. About 10% of patients receiving subcutaneous therapy were allowed to collect their medicine without a follow-up visit. Finally, 10% of patients living outside the Lazio region requested access to their therapy at a local centre closer to their home. At present, five patients have been found to be positive for SARS-CoV-2 infection but with minimal symptoms, 22 are in ‘quarantine’ for contact considered to be ‘at risk’ for the infection. Up to now, none of them has experienced significant symptoms. This study represents the first observational detailed report about short-term impact of the COVID-19 pandemic on patient organisation and management in a high-volume IBD centre.

## Introduction

Since January 2020 (December 2019 in China), coronavirus disease 2019 (COVID-19) caused by severe acute respiratory syndrome coronavirus 2 (SARS-CoV-2) infection has represented a dramatic public-health emergency both in Italy and worldwide.^1^,^2^

The high rate of transmission, frequent need for hospitalisation among symptomatic cases (∼10%), high requirement for intensive-care management and prominent mortality^1^,^2^ have led to a rapid change in the organisation of the health system at local as well as national and international levels. By mid April, Italy was the most affected country in Europe (more than 115,000 cases), with 55% of confirmed cases requiring hospitalisation, up to 10% requiring admission to intensive care units and a mortality rate close to 10%.^3^ Consequently, a governmental regulation on 4 March 2020 established that the entire country be considered a ‘red zone’, limiting non-essential activities and promoting confinement at home.

Accordingly, the logistical organisation of several hospitals all over the country has changed significantly, with huge investment in medical and non-medical resources in order to deal with this emergency. Moreover, to reduce the risk of spreading SARS-CoV-2 infection, access to hospitals has been strictly limited to urgent cases. In this context, the management of chronic diseases, including inflammatory bowel disease (IBD), has been critically impacted, with a significant reduction in visits, endoscopic and radiological procedures and multidisciplinary evaluations, modifying the current standard of care for IBD.^4^,^5^ There are currently only a few reports on IBD patients,^6–10^ but no specific recommendations can be given to IBD patients based on direct evidence.

The aim of this study was to give a report on major clinical and organisational challenges of a high-volume Italian hospital IBD centre a few months after the COVID-19 outbreak started and one month after the Italian government’s restrictions were imposed.

## Methods

### Study design

This was an observational prospective study reporting major clinical and organisational changes at the CEMAD (CEntro Malattie Apparato Digerente – Digestive Disease Centre) IBD Centre of the Fondazione Policlinico ‘A. Gemelli’ IRCCS, Rome, Italy, from 4 March 2020 to 15 April 2020.

IBD patients receiving biotechnological drugs or enrolled in clinical trials and who were regularly followed up at the CEMAD IBD Centre were included in the study. At the scheduled visit/therapy infusion, an evaluation on the outcome of access at the centre was provided for each patient. In particular, the access was considered ‘confirmed’ if no modification was observed, ‘delayed for clinical reasons’, ‘delayed due to patient choice’, ‘temporarily stopped’ or ‘rearranged at a closer IBD centre’ when therapy was performed at a centre closer to the patient’s home. Patient hospitalisations, infections, surgical interventions, adverse events or start of new therapy were also recorded.

### Physicians, nurses and other personnel involved in the study

The work plans of all medical and nonmedical staff (physicians, nurses and administrative personnel at the CEMAD IBD Centre were considered in the analysis. ‘Smart working’, ‘telemedicine’, changes in shifts or work attributed to a COVID or non-COVID path were registered. Physician and IBD nurse schedules and tasks were organised similarly to that stated by Fiorino et al.^5^

### Statistics

Results are reported as the mean ± standard deviation for continuous variables with a normal distribution, while discrete variables are expressed in percentages.

### Ethical considerations

The protocol was approved by the local Ethics Committee (Fondazione Policlinico Universitario ‘A. Gemelli’, 31 March 2020), and all enrolled patients gave written informed consent. The study protocol conforms to the ethical guidelines of the 1975 Declaration of Helsinki as reflected in prior approval by the Human Research Committee of the Fondazione Policlinico Universitario ‘A. Gemelli’.

## Results

A total of 1451 IBD patients on biological drugs or enrolled in clinical trials were included in the study. Their main clinical characteristics are summarised in Table 1.

**Table 1.** Patient characteristics.

Patients included, *N*	1451
Female, *n* (%)	609 (42)
Age (years), mean (*SD*)	44 (15)
Type of disease, *n* (%)	
Ulcerative colitis	522 (36)
Crohn’s disease	784 (54)
IBD-U	87 (6)
Pouchitis	87 (6)
Therapy, *n* (%) – already optimised, *n* (%)	
Infliximab	392 (27)–151 (39)
Adalimumab	450 (31)–213 (47)
Golimumab	44 (3)–23 (52)
Vedolizumab	218 (15)–111 (51)
Ustekinumab	131 (9)–94 (72)
Clinical trials	58 (4)–not applicable
Optimisation rate of biologicals during observation period	29 (2)
Temporary stopped before the COVID-19 pandemic or under review	169 (11)

*SD*: standard deviation; IBD-U: inflammatory bowel disease – unclassified; COVID-19: coronavirus disease 2019.

### Hospital reorganisation

**Figure 1.**
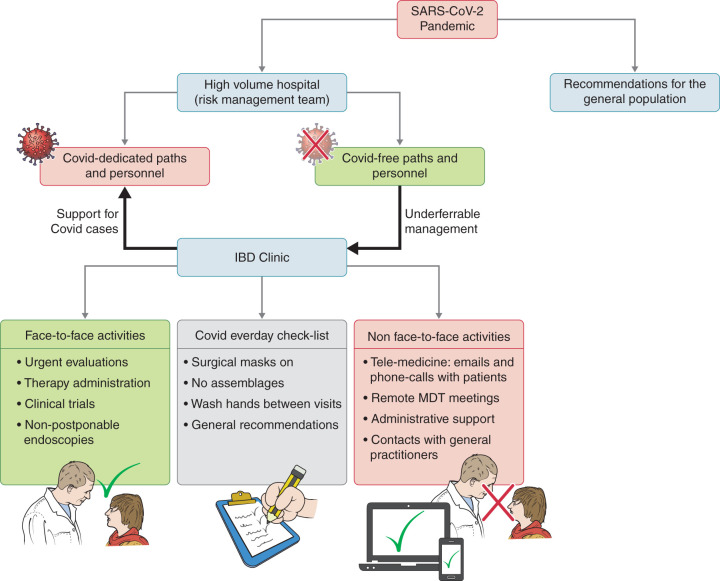
Reorganisation of the IBD centre within a high-volume hospital following the COVID-19 pandemic. The hospital’s risk-management team organised COVID paths, with dedicated personnel in the emergency department and on the ward. Every service, including laboratory, radiology, endoscopy, was reorganised in order to dedicate specific rooms or paths to COVID patients. All procedures were guided by the risk-management team, which was also supported by a panel of experts forming the hospital’s COVID Task Force. IBD organisation was maintained with everyday assessment in order to reduce the increase in SARS-CoV-2 infection. IBD: inflammatory bowel disease; COVID-19: coronavirus disease 2019; SARS-CoV-2: severe acute respiratory syndrome coronavirus 2.

Based on the experience in China, the hospital’s risk-management team organised a dedicated COVID path with dedicated personnel, including physicians and nurses at the emergency department and on the ward (Figure 1). Every service, including laboratory, radiology and endoscopy, was reorganised to dedicate specific rooms or paths to COVID patients. All procedures were guided by the risk-management team, which was also supported by a panel of experts forming the hospital’s COVID Task Force. All visits and access to the hospital that were clinically judged as not essential or which were deemed to be postponable were cancelled.

Everyone attending the hospital (physicians, nurses, administrative personnel, patients, etc.) were advised to respect the general recommended rules for hand hygiene and social distancing, and to disclose if they had a fever or cough or flu-like symptoms and to wear a surgical mask and gloves.^11^

### Reallocation of physicians, nurses and other IBD team members

With the progressive increase in COVID-positive patients attending the hospital, 40% (4/10) of physicians, 70% (5/7) of endoscopists and 50% of ultrasonographists dedicated to IBD services were shifted to inpatient COVID management. Up to 33% of fellows in IBD rotation were also reallocated to a COVID ward. Moreover, 30% of the administrative personnel dedicated to IBD outpatients were allowed to do ‘smart working’, with remote access to the Intranet system. Conversely, all dedicated IBD nurses, except for one considered at high risk, were maintained on their regular schedules.

### Changes to physicians’, nurses’ and administrative personnel’s work plans

All routine follow-up visits at the IBD centre for stable patients were cancelled or rescheduled for up to 12 weeks’ time. Up to 95% of multidisciplinary evaluations (including rheumatological, dermatological and nutritional, which are usually provided at the centre) were postponed. Proctological and surgical evaluations were maintained only for selected cases that were not considered deferrable according to clinical judgement. All access for biological therapies, clinical trials and unstable/relapsing patients were a priori considered non-postponable.

As far as trials are concerned, new screening and enrolment visits decreased by up to 50%, as several trials closed enrolment due to sponsor decision or because active monitoring on the site was not allowed by the clinical research organisation due to legislative limitations, and conversion in remote-controlled activities was not allowed. Furthermore, for trials involving oral or subcutaneous drugs, in cases where stable patients did not require medical follow-up visits, some trial sponsors arranged home delivery of the experimental drug.

**Figure 2.**
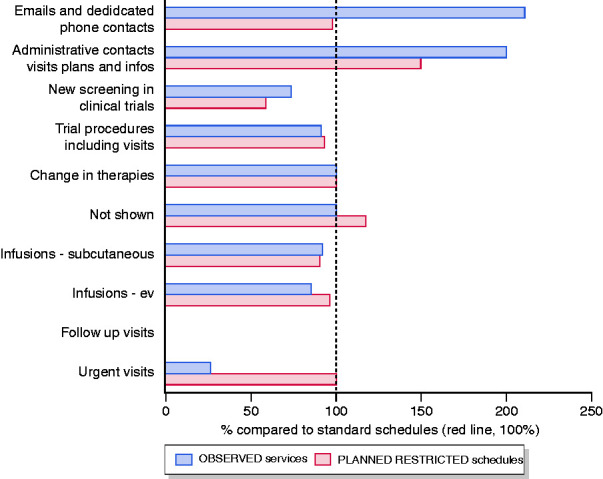
Work plan schedule changes before and after the COVID-19 pandemic at the IBD centre. Changes in planned and observed schedules are expressed as percentages over standard schedules. Calculations are made based on weekly schedules: standard – planned – observed activities (absolute numbers rounded off to the nearest unit). Email and phone contacts: 142 – 140 – 300; administrative contacts: 300 – 450 – 600; new screening in clinical trials: 4 – 2 – 3; trial procedures: 12 – 11 – 11; changes in therapies: 3 – 3 – 3; not shown: 9 – 10 – 9; infusions – subcutaneous: 55 – 50 – 51; infusions – iv (intravenous): 91 – 90 – 80; follow-up visits: 127 – 0 – 0; urgent visits: 15 – 15 – 4.

A significant change to the actual work plan was observed. Figure 2 shows the percentages of observed versus expected activities. In particular, no follow-up visits for patients in sustained remission were performed during the chosen time frame. However, when rescheduling the visits, patients were advised to disclose potential symptoms indicating IBD reactivation in order to confirm that the visit was actually postponable. Furthermore, they were invited to contact the clinic by mail in four to six weeks (or less if necessary) in order to provide an update on their well-being. We observed lower than expected rates of (a) urgent visits (50% decrease compared to the standard plans), (b) infusion procedures and (c) access of patients on subcutaneous biological treatment. Notably, in our hospital, subcutaneous drugs are usually dispensed by dedicated IBD nurses (and not by hospital pharmacist) in order to keep track of the patient and to control therapy compliance better. Furthermore, at the beginning of April, Lazio region promoted a legislative initiative to allow the home delivery of subcutaneous drugs for patients not requiring any medical advice or follow-up visits.

The number of patients who did not show up for scheduled visits increased, but less than expected. Conversely, email and phone contact increased by more than 200%, being the major drivers for change in physicians’ work plans. Informative phone calls or emails were the major change drivers for administrative staff.

Currently, no major work overload for physicians and nurses has been observed. However, this needs to be checked frequently. Being a university hospital, an increase in workload has been observed for health-care professionals with university positions in order to provide online classes for medical students.

So far, no quarantine, no infections and no respiratory symptoms have been observed among health-care professionals, whose state of health was self-assessed every day. Operational meetings have been maintained if the general safety recommendations could be respected. All important meetings have been also web based in order to minimise people congregating.

### Patients

**Figure 3.**
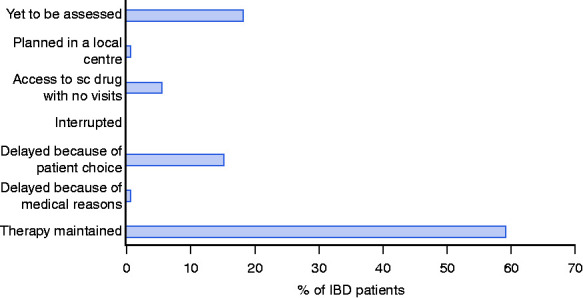
Patients under biological and experimental therapy management after COVID-19 pandemic at the IBD centre. Absolute numbers (and percentages) of observed cases over the general IBD population receiving biological or clinical trial therapy are shown. Out of 1451 patients, 266 (18.3%) were yet to be assessed, 11 (0.77%) were planned in a local centre, 82 (5.62%) had access to subcutaneous drugs with no follow-up visit, 0 (0%) were interrupted, 222 (15.33%) were delayed because of patient's choice, 11 (0.77%) were delayed because for medical reasons and 859 (59.2%) had their therapy maintained.

To date, more than half of the patients have been assessed at the time of the scheduled visit, while the rest, expected in the following weeks, have been contacted by phone or email to have a preventive confirmation of the scheduled visit (Figure 3). About 65% of patients maintained their schedules without major problems, 20% experienced a delay to the planned infusions because of the patient’s decision or for practical issues. About 10% of patients on subcutaneous therapy were confirmed for therapy and were able to collect their drugs without follow-up appointment. A very limited number of patients, nearly 10%, asked for their therapy to be moved to a centre closer to their home. At the last evaluation, five patients receiving biological/immunosuppressive therapy were found to be positive for COVID-19 infection, 22 patients were in ‘quarantine’ for contact considered to be ‘at risk’ for infection and no one experienced severe COVID-19 symptoms. Furthermore, compared to the same period last year, no increase in hospitalisation rates or need for multidisciplinary evaluations was observed (data not shown). Although new inductions of biological therapy were authorised, the number of new inductions decreased compared to the same period last year (data not shown). Finally, even though psychological support was regularly provided as standard at the CEMAD IBD centre through a telephone helpline or patient organisations, no major problems were registered on this topic for the vast majority of patients. Of note, the only death registered was for the suicide of a psychotic patient outside the Lazio region.

## Discussion

This study represents the first detailed observational report about the short-term impact of the COVID-19 pandemic on IBD organisations and its management in an Italian IBD referral centre (Figure 1). The report focuses prominently on hospital organisation, personnel management and work plans. The logistic rearrangements were driven by the risk-management team, which has been the main guide for the hospital during this historic period. At the same time, we have tried to organise our work plans in accordance with current clinical necessity.

At one-month follow-up, we experienced a significant increase in email and phone contacts for both physicians and administrative personnel. No infections have been registered so far among the personnel attending IBD clinic, and quarantine has not been necessary. None of the clinical personnel have been checked for active or subclinical SARS-CoV-2 infection. This issue could generate some debate, considering a relatively high prevalence of SARS-CoV-2 infection among health-care professionals in other clinical settings.^12^ However, we can assume that our specific setting is potentially characterised by a lower prevalence of SARS-CoV-2 infection: COVID-dedicated paths and strict prophylactic measures promptly established by the hospital (with personal protective equipment accessible for all personnel and patients) might have helped to prevent the spread of the infection among our clinical staff.

Furthermore, our work focused on IBD patients attending the centre to receive biological therapy and/or innovative clinical trial drugs. IBD patients’ reacted to the emergency situation by apparently coping positively with coronavirus, without any evident increase in request for psychological support. Patients’ main concerns were about COVID-19 disease and the possible impact of biological therapies on SARS-CoV-2 infection. Most of our patients directly asked, through emails or phone calls, if they needed to stop biological therapy in order to reduce their risk for COVID-19 disease. Press and social media really stressed the concept that so-called ‘immunocompromised’ patients were indeed at greater risk for COVID-19 disease and complications. Our general recommendations, in accordance with the statements coming from the International Organization for the Study of Inflammatory Bowel Diseases (IOIBD),^10^ were that all IBD maintenance therapies needed to continue. To date, no evidence exists regarding a drugs-related increased risk of SARS-CoV-2 infection for IBD patients. Conversely, disease reactivation, requiring hospitalisation and intensive treatment, due to drug withdrawal is a greater concern. Furthermore, dehydration and anaemia secondary to active disease could potentially worsen a patient’s outcome in the case of SARS-CoV-2 infection.

According to IOIBD recommendations, three classes of drugs might represent a potential issue in relation to SARS-CoV-2 infection: thiopurines, corticosteroids and Janus kinase (JAK) inhibitors.^10^ Thiopurines, despite being associated with an increased risk of viral infections,^13^ should be not withdrawn to prevent SARS-CoV-2 infection, since they continue to exert their immunosuppressive effects for several months after discontinuation. So, their cessation would not provide any immediate benefit.^14^ As far as steroids are concerned, we can broadly say that our prescriptions have not affected by the outbreak of COVID-19, when strictly necessary. On the other hand, considering the warning of an increased risk of SARS-CoV-2 infection and COVID-19 disease for a dose >20 mg/day of prednisone,^13^ closer attention has been paid to steroid tapering in order to reduce exposure to high doses.^10^ Finally, JAK inhibitors are not currently reimbursed for IBD patients in Italy.

Despite the general recommendations of IBD specialists, up to 10% of patients nonetheless decided to delay or postpone biological therapy, suggesting a high level of attention of IBD patients to potential immunomodulator activity and the impact of IBD-related drugs on SARS-CoV-2 infection susceptibility. No short-term clinical impact was observed.

This study does not show any data regarding mild IBD population not under biological/experimental therapies. This is a population usually attending the hospital outpatient service which cannot be properly considered an immunocompromised population. All patients who expected to attend the centre until the end of April 2020 were contacted by the administrative personnel who postponed the visits. Furthermore, all patients could benefit from direct contact with the IBD centre in the case of reactivation or special needs. We can presume that a low prevalence of SARS-CoV-2 infection can also be observed in this category of patients up to now, as no patients have reported any contact with a SARS-CoV-2-positive patient with either active COVID-19 disease or suspicion of it.

This study has several limitations. First, the relatively short follow-up period could mean the effect of the pandemic on the long-term organisation of the IBD clinic within the hospital has been underestimated. Moreover, no further information can be given on the impact of the pandemic on disease severity, psychological distress of patients or even on the prevalence and severity of SARS-CoV-2 infection in IBD patients under biological therapy. To date, there are no data in favour of or against monoclonal antibodies, although anti-interleukin-6 agents appear to be promising for the treatment of COVID-19 pneumonia. Based on our judgement and on international society recommendations,^10^ until dedicated data are available, treatment continuation should be advised.

This report strongly suggests that optimising access to the IBD unit and implementing official telemedicine and remote communications between patients and physicians could perhaps reduce the presence of IBD patients within the hospital and reduce the potential disservice to these patients produced by an unexpected pandemic, with little, if any, impact on the quality of the health-care provided to those patients.

Finally, we can assume that correct management of IBD patients, based on clear communication and reassurance, might positively impact their outcomes. In the vast majority of cases, therapies have been continued, thus avoiding relapses, and unnecessary hospital access has been prevented.
